# Free Vibration Analysis of Curved Laminated Composite Beams with Different Shapes, Lamination Schemes, and Boundary Conditions

**DOI:** 10.3390/ma13041010

**Published:** 2020-02-24

**Authors:** Bin Qin, Xing Zhao, Huifang Liu, Yongge Yu, Qingshan Wang

**Affiliations:** 1 Key Laboratory of Traffic Safety on Track, Ministry of Education, School of Traffic & Transportation Engineering, Central South University, Changsha 410075, China; qinbin@csu.edu.cn (B.Q.); liuhf_csu@163.com (H.L.); 2 Joint International Research Laboratory of Key Technology for Rail Traffic Safety, Central South University, Changsha 410075, China; 3 National & Local Joint Engineering Research Center of Safety Technology for Rail Vehicle, Central South University, Changsha 410075, China; 4 State Key Laboratory of High Performance Complex Manufacturing, Central South University, Changsha 410083, China; qingshanwang@csu.edu.cn; 5 Overall R&D Department, CRRC Changchun Railway Vehicles Co. LTD, Changchun 130062, China; yuyongge.a@cccar.com.cn

**Keywords:** modified variational approach, curved laminated composite beams, Jacobi polynomials, alterable curvatures, multi-segment partitioning strategy

## Abstract

A general formulation is considered for the free vibration of curved laminated composite beams (CLCBs) with alterable curvatures and diverse boundary restraints. In accordance with higher-order shear deformation theory (HSDT), an improved variational approach is introduced for the numerical modeling. Besides, the multi-segment partitioning strategy is exploited for the derivation of motion equations, where the CLCBs are separated into several segments. Penalty parameters are considered to handle the arbitrary boundary conditions. The admissible functions of each separated beam segment are expanded in terms of Jacobi polynomials. The solutions are achieved through the variational approach. The proposed methodology can deal with arbitrary boundary restraints in a unified way by conveniently changing correlated parameters without interfering with the solution procedure.

## 1. Introduction

The moderately tall curved laminated composite beams (CLCBs) are widely applied in engineering fields. Besides, the laminated composite material has excellent mechanical properties including high compression-resistance capacity, high strength-to-weight and stiffness-to-weight ratios, preeminent corrosion-resistance, and powerful customizable capacity [1,2]. In practical use, the CLCBs may commonly undergo various kinds of complicated dynamic loads and other complex work environments, which lead to excessive vibration and fatigue damage of the structure. Dynamic modelling is the precondition for understanding the vibration characteristics of CLCBs. Thus, this paper aims to evaluate the vibration features of CLCBs with alterable curvatures by a modified variational approach in the framework of higher-order shear deformation theory (HSDT). 

Numerous works have been carried out on the vibration problems of CLCBs. A group of equations were constructed by Qatu [3] for vibration analysis of simply supported CLCBs, which are thin or moderately tall. The influences of rotary inertia, shear deformation, thickness ratio, material orthotropy, and curvature on the vibration frequencies were investigated. Utilizing the Ritz method, Qatu [4,5] implemented dynamic analysis of CLCBs with shallow and deep curvatures. The in-plane vibration characteristics were investigated. By the dynamic stiffness approach in conjunction with series solutions, vibration analysis of curved laminate beams was conducted by Tseng et al. [6]. The impacts of rotary inertia, as well as shear deformation, were taken into account for the numerical model. A novel layer-wise displacement model was put forward by Carpentieri et al. [7] to capture the dynamic behaviors of CLCBs subjected to classic restraints. In the framework of shallow shell theory assumptions, a modal method was proposed by Khdeir and Reddy [8] to unearth the dynamic features of a shallow laminated composite arch subjected to various boundary restraints. Surana and Nguyen [9] proposed a two-dimensional (2D) element formulation for the dynamic analysis of a curved beam by utilizing HSDT. Exploiting first-order shear deformation theory (FSDT), the dynamic behavior of simply supported CLCBs with deep curvatures was studied by Hajianmaleki and Qatu [10]. The general differential quadrature method (DQM) was adopted for the formulation. The vibration features of curved delaminated composite beams were studied by Jafari et al. [11] via a finite element approach and analytical solution. The influences of rotary inertia, material coupling, shear deformation, and the deepness term were taken into consideration. On the foundation of 2D elasticity theory, the vibration features of CLCBs were investigated by Chen et al. [12], where the state space approach and DQM method were employed. A one-dimensional (1D) mechanical model was considered by Ascione and Fraternali [13] for dynamic analysis of CLCBs. The model was constructed based on the stationary potential energy theory, and penalty terms were employed to impose the restraint conditions. Only classic boundary conditions were taken into account. The vibration features of deep CLCBs subjected to various boundary conditions were analyzed by Ye et al. [14] through the Ritz method, where the improved Fourier series was used for the admissible displacement functions. Based on NURBS (Non-Uniform Rational B-Splines), isogeometric analysis was implemented by Luu et al. [15] for understanding the dynamic behaviors of Timoshenko-type deep curved laminated beams with various curvatures. The instantaneous responses of curved composite laminated beams were studied by Shao et al. [16], where the reverberation ray matrix approach was introduced. The curved beams were subjected to arbitrary boundary restraints and diverse lamination schemes were considered. A general and unified formulation was proposed by Qu et al. [17] for vibration analysis of CLCBs subjected to diverse boundary restraints. The modified variational principle was used for the derivation of the formulation, and a multi-segment partitioning strategy was employed. The vibration characteristics of glass fiber-reinforced polymer (GFRP) composites reinforced with nylon nano-fibers were experimentally and numerically studied by Garcia et al. [18,19]. Recently, the spectral-Tchebychev technique, which utilizes Tchebychev polynomials for the spatial discretization, has been proposed by several researchers [20,21,22]. The method incorporates different boundary conditions by projection matrices, where solutions for various linear and nonlinear vibration problems with different boundary conditions can be easily obtained.

The development of calculating methods of high precision has become an important subject, and multitudinous beam theories and calculation methods have been developed on the foundation of diverse assumptions and approximations. From a literature review, it has been found that most previous investigations about the free vibration of CLCBs have been confined to classical boundary restraints. For accurate beam theories, three major categories are adopted, commonly including classical, first-order, and higher-order beam theories. The classical beam theory (CBT) is suitable for slender beams. As the influence of the transverse shear deformation is ignored by CBT, the deflection is underestimated and the natural frequency is overestimated for tall beams. To make up for this deficiency, the first-order beam theory (FBT) was developed. For FBT, a shear correction factor is introduced to express the transverse shear force, the values of which are affected by a lot of parameters (e.g., layer sequences, material properties, etc.) and which will significantly affect the accuracy of the results. To further improve and perfect the beam theory, the HSDT was developed. Compared to CBT and FBT, HSDT can express the kinematics of the CLCB better and can produce more precise interlaminar stress distributions. Besides, the shear deformation effects are considered for HSDT, which includes the correct cross-sectional warping without the requirement of a shear correction factor. However, HSDT involves higher-order stress resultants and, thus, needs a higher computational cost, which is one limitation of the present approach.

In addition, the majority of studies have been conducted on the vibration features of circular CLCBs, while those of others are rare. The reason for this is that when the curvature radius is changed, the more complex description of the geometry will make the solution become very difficult [23]. On the whole, investigations of the free vibration of CLCBs subjected to arbitrary boundary restraints and with alternate curvatures are limited. This paper proposes a general and unified formulation for vibration analysis of CLCBs with various curvatures. The novelties of the present paper are as follows: (1) First, most of the previous studies concerned curved circular composite laminated beams (Qu et al. [17]), while studies of CLCBs with various curvatures are rare; (2) a variational method in conjunction with a multi-segment partitioning strategy and HSDT for CLCBs is presented, which has the following advantages: (a) Allowing flexible choices of admissible functions, (b) fast and stable convergence characteristics, and (c) high accuracy, especially for higher modes; (3) the penalty and boundary parameters are incorporated for the convenient change of boundary conditions including classic and elastic ones. On the whole, this approach should be valuable in both theoretical and engineering aspects. The outline of the paper is divided into three parts: (1) Theoretical formulations; (2) results and discussions; and (3) conclusions. 

## 2. Theoretical Formulations

### 2.1. Description of the CLCB Model

**Figure 1:**
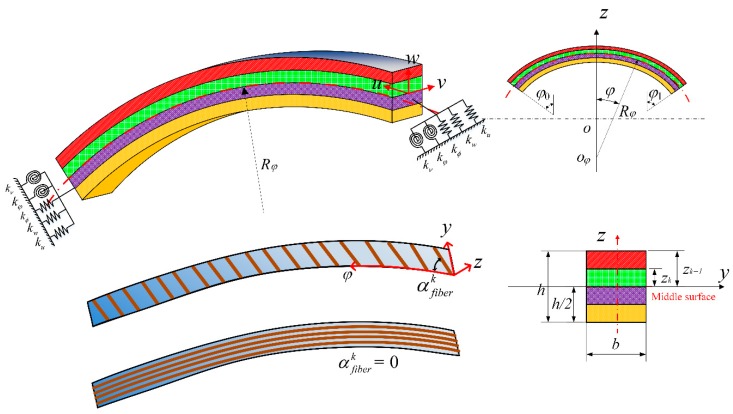
Schematic plot and geometric parameters of curved composite laminated beams.

**Figure 2:**
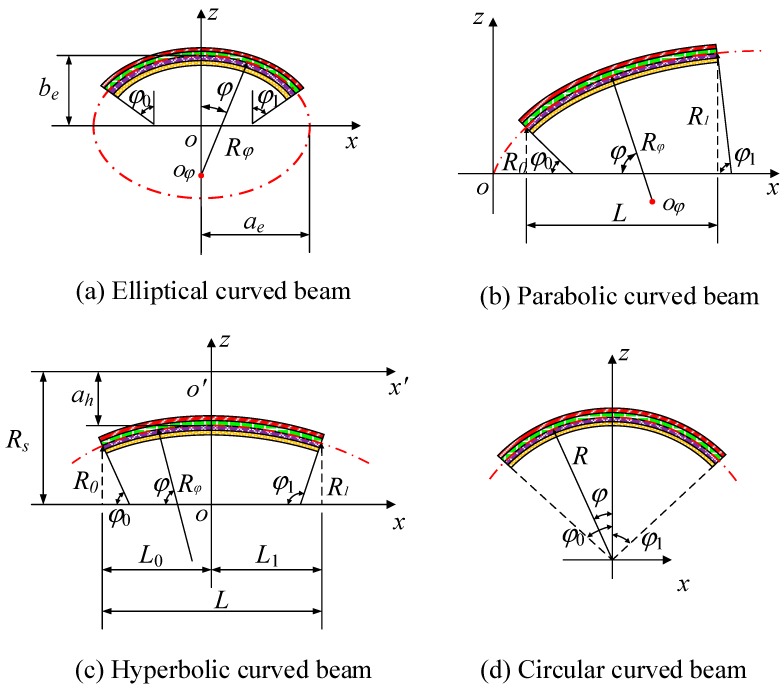
Different types of curved laminated composite beams (CLCBs): (**a**) Elliptic curved beam; (**b**) parabolic curved beam; (**c**) hyperbolic curved beam; (**d**) circular curved beam.

The schematic plot and geometric parameters of a CLCB are presented in Figure 1. The curved beam has curvature radius *R_φ_*, thickness *h*, and width *b*. The orthogonal coordinates (*φ*, *y*, *z*) are located at the middle surface of the CLCB. The *φ*-, *y*-, and *z*- coordinates are along the central line (indicated as red line), width, and thickness orientations, respectively. The curved beam comprises several orthotropic layers, where *Z_k_* represents the distance from the top surface of the *k*’th layer to the middle surface. The fiber orientation angle of the *k*’th lamina with respect to the *φ*-axis of the CLCBs is defined as $\alpha_{fiber}^{k}$, and the case of $\alpha_{fiber}^{k}$ = 0 is presented for a better understanding. Vibration of the CLCB occurs in the *φ–z* plane and can be characterized by the central line. In engineering applications, different types of CLCBs with diverse curvatures may be encountered, e.g., hyperbolic, parabolic, elliptic, and circular ones. For each type, their radii of curvatures $R_{\varphi }(\varphi )$ can be described in terms of different geometric parameters. Different types of CLCBs are shown in Figure 2; note that the *x*-axis, which is perpendicular to the *z*-axis in the *φ–z* plane, is added for a better description.

For an elliptic curved beam (Figure 2a), $R_{\varphi }(\varphi )$ is defined in terms of semimajor axis length *a_e_* and semiminor axis length *b_e_*:

(1) $$R_{\varphi }(\varphi )=\frac{a_{e}^{2}b_{e}^{2}}{\sqrt{{\left(a_{e}^{2}\sin^{2}\varphi +b_{e}^{2}\cos^{2}\varphi \right)}^{3}}}.$$

For a parabolic curved beam (Figure 2b), $R_{\varphi }(\varphi )$ can be expressed as

(2) $$R_{\varphi }(\varphi )=\frac{k}{2\cos^{3}\varphi }\;,k=\frac{R_{1}^{2}-R_{0}^{2}}{L},\;\varphi_{0}=\arctan\left(\frac{2R_{0}}{k}\right),\;\varphi_{1}=\arctan\left(\frac{2R_{1}}{k}\right),$$

where *R*_0_ and *R*_1_ indicate the horizontal radius. 

The hyperbolic CLCB (Figure 2c) is represented by the following equations:

(3a) $$R_{\varphi }(\varphi )=\frac{-a_{h}^{2}b_{h}^{2}}{\sqrt{{\left(a_{h}^{2}\sin^{2}\varphi -b_{h}^{2}\cos^{2}\varphi \right)}^{3}}},$$

(3b) $$b_{h}=L_{0}a_{h}/\sqrt{\left(R_{0}^{2}-a_{h}^{2}\right)}=L_{1}a_{h}/\sqrt{\left(R_{1}^{2}-a_{h}^{2}\right)},$$

(3c) $$\varphi_{0}=\arctan\left(\frac{\left(R_{0}-R_{s}\right)b_{h}}{a_{h}\sqrt{{\left(R_{0}-R_{s}\right)}^{2}-a_{h}^{2}}}\right),$$

(3d) $$\varphi_{1}=\pi -\arctan\left(\frac{\left(R_{1}-R_{s}\right)b_{h}}{a_{h}\sqrt{{\left(R_{1}-R_{s}\right)}^{2}-a_{h}^{2}}}\right),$$

where *R_s_* denotes the distance from *ox* to $o^{\prime }x^{\prime }$(revolution axis); *a_h_* signifies the length of the semitransverse axis, and *b_h_* indicates that of the semiconjugate axis.

Figure 2d shows the circular curved beam, where *R* indicates the mean radius.

### 2.2. Energy Expression of the CLCB 

The energy expressions of the CLCB are established in the framework of HSDT. Hence, the displacement components of the CLCB for an arbitrary point can be written as

(4a) $$\bar{U}\left(\varphi ,y,z,t\right)=u\left(\varphi ,y,t\right)+\psi_{\varphi }\left(\varphi ,y,t\right)z+\phi_{\varphi }\left(\varphi ,y,t\right)z^{2}+\lambda_{\varphi }\left(\varphi ,y,t\right)z^{3},$$

(4b) $$\bar{W}\left(\varphi ,y,z,t\right)=w\left(\varphi ,y,t\right),$$

in which *u* and *w* indicate the displacements along the *φ* and *z* orientations at the middle surface, respectively. In addition, $\psi_{\varphi }$ denotes the rotation of the transverse normal with respect to the *φ* direction. $\phi_{\varphi }$ and $\lambda_{\varphi }$ signify the higher-order terms connected to the Taylor series. Mathematically,

(5) $$\psi_{\varphi }={\left(\frac{\partial \bar{U}}{\partial z}\right)}_{z=0},\phi_{\varphi }=\frac{1}{2}{\left(\frac{\partial^{2}\bar{U}}{\partial z^{2}}\right)}_{z=0},\lambda_{\varphi }=\frac{1}{6}{\left(\frac{\partial^{3}\bar{U}}{\partial z^{3}}\right)}_{z=0}.$$

The linear strain–displacement relationships, which consider *z*/*R_φ_* terms, can be given as follows:

(6a) $$\varepsilon_{\varphi }=\frac{1}{1+z/R_{\varphi }}\left(\varepsilon_{\varphi }^{0}+zk_{\varphi }^{0}+z^{2}k_{\varphi }^{1}+z^{3}k_{\varphi }^{2}\right),$$

(6b) $$\gamma_{\varphi z}=\frac{1}{1+z/R_{\varphi }}\left(\gamma_{\varphi z}^{0}+zk_{\varphi z}^{0}+z^{2}k_{\varphi z}^{1}+z^{3}k_{\varphi z}^{2}\right),$$

where $\varepsilon_{\varphi }^{0}$ and $\gamma_{\varphi z}^{0}$ signify the middle surface normal and shear strains, respectively, and $k_{\varphi }^{i(i=0,1,2)}$ and $k_{\varphi z}^{i(i=0,1,2)}$ indicate curvature changes. They are defined as

(7a) $$\varepsilon_{\varphi }^{0}=\frac{1}{R_{\varphi }}\left(\frac{\partial u}{\partial \varphi }+w\right),k_{\varphi }^{0}=\frac{1}{R_{\varphi }}\frac{\partial \psi_{\varphi }}{\partial \varphi },k_{\varphi }^{1}=\frac{1}{R_{\varphi }}\frac{\partial \phi_{\varphi }}{\partial \varphi },k_{\varphi }^{2}=\frac{1}{R_{\varphi }}\frac{\partial \lambda_{\varphi }}{\partial \varphi },$$

(7b) $$\gamma_{\varphi z}^{0}=\frac{1}{R_{\varphi }}\left(-u+\frac{\partial w}{\partial \varphi }\right)+\psi_{\varphi },k_{\varphi z}^{0}=2\phi_{\varphi },k_{\varphi z}^{1}=\frac{1}{R_{\varphi }}\frac{\partial \phi_{\varphi }}{\partial \varphi }+3\lambda_{\varphi },k_{\varphi z}^{2}=2\frac{1}{R_{\varphi }}\lambda_{\varphi }.$$

The force (*N*) and moment resultants (*M*) of the CLCB can be represented by middle surface strains and curvature changes:

(8a) $$\left\{\begin{array}{l} N_{\varphi }\\ N_{y}\\ N_{\varphi y}\\ M_{\varphi }\\ M_{y}\\ M_{\varphi y}\\ N_{\varphi }^{1}\\ N_{y}^{1}\\ N_{\varphi y}^{1}\\ M_{\varphi }^{2}\\ M_{y}^{2}\\ M_{\varphi y}^{2} \end{array}\right\}=\left[\begin{array}{cccc} \begin{array}{ccc} A_{11} & A_{12} & A_{16}\\ A_{12} & A_{22} & A_{26}\\ A_{16} & A_{26} & A_{66} \end{array} & \begin{array}{ccc} B_{11} & B_{12} & B_{16}\\ B_{12} & B_{22} & B_{26}\\ B_{16} & B_{26} & B_{66} \end{array} & \begin{array}{ccc} C_{11} & C_{12} & C_{16}\\ C_{12} & C_{22} & C_{26}\\ C_{16} & C_{26} & C_{66} \end{array} & \begin{array}{ccc} D_{11} & D_{12} & D_{16}\\ D_{12} & D_{22} & D_{26}\\ D_{16} & D_{26} & D_{66} \end{array}\\ \begin{array}{ccc} B_{11} & B_{12} & B_{16}\\ B_{12} & B_{22} & B_{26}\\ B_{16} & B_{26} & B_{66} \end{array} & \begin{array}{ccc} C_{11} & C_{12} & C_{16}\\ C_{12} & C_{22} & C_{26}\\ C_{16} & C_{26} & C_{66} \end{array} & \begin{array}{ccc} D_{11} & D_{12} & D_{16}\\ D_{12} & D_{22} & D_{26}\\ D_{16} & D_{26} & D_{66} \end{array} & \begin{array}{ccc} E_{11} & E_{12} & E_{16}\\ E_{12} & E_{22} & E_{26}\\ E_{16} & E_{26} & E_{66} \end{array}\\ \begin{array}{ccc} C_{11} & C_{12} & C_{16}\\ C_{12} & C_{22} & C_{26}\\ C_{16} & C_{26} & C_{66} \end{array} & \begin{array}{ccc} D_{11} & D_{12} & D_{16}\\ D_{12} & D_{22} & D_{26}\\ D_{16} & D_{26} & D_{66} \end{array} & \begin{array}{ccc} E_{11} & E_{12} & E_{16}\\ E_{12} & E_{22} & E_{26}\\ E_{16} & E_{26} & E_{66} \end{array} & \begin{array}{ccc} F_{11} & F_{12} & F_{16}\\ F_{12} & F_{22} & F_{26}\\ F_{16} & F_{26} & F_{66} \end{array}\\ \begin{array}{ccc} D_{11} & D_{12} & D_{16}\\ D_{12} & D_{22} & D_{26}\\ D_{16} & D_{26} & D_{66} \end{array} & \begin{array}{ccc} E_{11} & E_{12} & E_{16}\\ E_{12} & E_{22} & E_{26}\\ E_{16} & E_{26} & E_{66} \end{array} & \begin{array}{ccc} F_{11} & F_{12} & F_{16}\\ F_{12} & F_{22} & F_{26}\\ F_{16} & F_{26} & F_{66} \end{array} & \begin{array}{ccc} G_{11} & G_{12} & G_{16}\\ G_{12} & G_{22} & G_{26}\\ G_{16} & G_{26} & G_{66} \end{array} \end{array}\right]\left\{\begin{array}{l} \varepsilon_{\varphi }^{0}\\ \varepsilon_{y}^{0}\\ \gamma_{\varphi y}^{0}\\ k_{\varphi }^{0}\\ k_{y}^{0}\\ k_{\varphi y}^{0}\\ k_{\varphi }^{1}\\ k_{y}^{1}\\ k_{\varphi y}^{1}\\ k_{\varphi }^{2}\\ k_{y}^{2}\\ k_{\varphi y}^{2} \end{array}\right\},$$

(8b) $$\left\{\begin{array}{l} Q_{\varphi }\\ Q_{y}\\ P_{\varphi }\\ P_{y}\\ Q_{\varphi }^{1}\\ Q_{y}^{1}\\ P_{\varphi }^{2}\\ P_{y}^{2} \end{array}\right\}=\left[\begin{array}{cccc} \begin{array}{cc} A_{55} & A_{45}\\ A_{45} & A_{44} \end{array} & \begin{array}{cc} B_{55} & B_{45}\\ B_{45} & B_{44} \end{array} & \begin{array}{cc} C_{55} & C_{45}\\ C_{45} & C_{44} \end{array} & \begin{array}{cc} D_{55} & D_{45}\\ D_{45} & D_{44} \end{array}\\ \begin{array}{cc} B_{55} & B_{45}\\ B_{45} & B_{44} \end{array} & \begin{array}{cc} C_{55} & C_{45}\\ C_{45} & C_{44} \end{array} & \begin{array}{cc} D_{55} & D_{45}\\ D_{45} & D_{44} \end{array} & \begin{array}{cc} E_{55} & E_{45}\\ E_{45} & E_{44} \end{array}\\ \begin{array}{cc} C_{55} & C_{45}\\ C_{45} & C_{44} \end{array} & \begin{array}{cc} D_{55} & D_{45}\\ D_{45} & D_{44} \end{array} & \begin{array}{cc} E_{55} & E_{45}\\ E_{45} & E_{44} \end{array} & \begin{array}{cc} F_{55} & F_{45}\\ F_{45} & F_{44} \end{array}\\ \begin{array}{cc} D_{55} & D_{45}\\ D_{45} & D_{44} \end{array} & \begin{array}{cc} E_{55} & E_{45}\\ E_{45} & E_{44} \end{array} & \begin{array}{cc} F_{55} & F_{45}\\ F_{45} & F_{44} \end{array} & \begin{array}{cc} G_{55} & G_{45}\\ G_{45} & G_{44} \end{array} \end{array}\right]\left\{\begin{array}{l} \gamma_{\varphi z}^{0}\\ \gamma_{yz}^{0}\\ k_{\varphi z}^{0}\\ k_{yz}^{0}\\ k_{\varphi z}^{1}\\ k_{yz}^{1}\\ k_{\varphi z}^{2}\\ k_{yz}^{2} \end{array}\right\},$$

where $N_{\varphi }$, *N_y_*, *N_φy_*, $N_{\varphi }^{1}$, $N_{y}^{1}$, and $N_{\varphi y}^{1}$ signify the in-plane forces; $M_{\varphi }$, *M_y_*, *M_φy_*, $M_{\varphi }^{2}$, $M_{y}^{2}$, and $M_{\varphi y}^{2}$ denote the moment resultants; and $Q_{\varphi }$, *Q_y_*, $P_{\varphi }$, $P_{y}$, $Q_{\varphi }^{1}$, $Q_{y}^{1}$, $P_{\varphi }^{2}$, and $P_{y}^{2}$ indicate the transverse shear force resultants. The corresponding extensional stiffnesses *A_ij_* (*i*, *j* = 1, 2, 6), coupling stiffnesses *B_ij_*, bending stiffnesses *C_ij_*, and other stiffness coefficients ($D_{ij},E_{ij},F_{ij},G_{ij}\;\mathrm{and}\;H_{ij}$) can be expressed as

(9) $$\left\{A_{ij},B_{ij},C_{ij},D_{ij},E_{ij},F_{ij},G_{ij},H_{ij}\right\}=\sum_{k=1}^{N_{k}}\int_{z_{k}}^{z_{k+1}}{\bar{S}}_{ij}^{k}\left\{1,z,z^{2},z^{3},z^{4},z^{5},z^{6},z^{7}\right\}dz,$$

where ${\bar{S}}_{ij}^{k}$ indicates transformed reduced stiffness coefficients for the *k*’th layer. In addition, they are defined as

(10) $${\bar{S}}_{11}^{k}=S_{11}^{k}m^{4}+2\left(S_{12}^{k}+2S_{66}^{k}\right)m^{2}n^{2}+S_{22}^{k}n^{4},\;{\bar{S}}_{12}^{k}=\left(S_{11}^{k}+S_{22}^{k}-4S_{66}^{k}\right)m^{2}n^{2}+S_{12}^{k}\left(m^{4}+n^{4}\right),\;{\bar{S}}_{22}^{k}=S_{11}^{k}n^{4}+2\left(S_{12}^{k}+2S_{66}^{k}\right)m^{2}n^{2}+S_{22}^{k}m^{4},\;{\bar{S}}_{16}^{k}=\left(S_{11}^{k}-S_{12}^{k}-2S_{66}^{k}\right)m^{3}n+\left(S_{12}^{k}-S_{22}^{k}+2S_{66}^{k}\right)mn^{3},\;{\bar{S}}_{26}^{k}=\left(S_{11}^{k}-S_{12}^{k}-2S_{66}^{k}\right)mn^{3}+\left(S_{12}^{k}-S_{22}^{k}+2S_{66}^{k}\right)m^{3}n,\;{\bar{S}}_{66}^{k}=\left(S_{11}^{k}+S_{22}^{k}-2S_{12}^{k}-2S_{66}^{k}\right)m^{2}n^{2}+S_{66}^{k}\left(m^{4}+n^{4}\right),\;{\bar{S}}_{44}^{k}=S_{44}^{k}m^{2}+S_{55}^{k}n^{2},\;{\bar{S}}_{45}^{k}=\left(S_{55}^{k}-S_{44}^{k}\right)mn,\;{\bar{S}}_{55}^{k}=S_{55}^{k}m^{2}+S_{44}^{k}n^{2},\;S_{11}^{k}=\frac{E_{1}^{k}}{1-\mu_{12}^{k}\mu_{21}^{k}},\;S_{12}^{k}=\frac{\mu_{12}^{k}E_{2}^{k}}{1-\mu_{12}^{k}\mu_{21}^{k}},\;S_{22}^{k}=\frac{E_{2}^{k}}{1-\mu_{12}^{k}\mu_{21}^{k}},\;S_{44}^{k}=G_{23}^{k},\;S_{55}^{k}=G_{13}^{k},\;S_{66}^{k}=G_{12}^{k},\;m=\cos\alpha_{fiber}^{k},n=\sin\alpha_{fiber}^{k},$$

where $\alpha_{fiber}^{k}$ denotes the fiber orientation angle of the *k*’th lamina with respect to the *φ*-axis of the CLCBs and $S_{ef}^{k}$ (*e*, *f* = 1, 2, 4, 5, and 6) is the material properties. With regard to CLCBs, the following parameters (*N_y_*, *N_φy_*, $N_{y}^{1}$, and $N_{\varphi y}^{1}$; *Q_y_*, $P_{y}$, $Q_{y}^{1}$, and $P_{y}^{2}$; *M_y_*, *M_φy_*, $M_{y}^{2}$, and $M_{\varphi y}^{2}$) are presumed to be zero. Hence, Equation (8) can be rewritten as

(11) $$\left\{\begin{array}{l} N_{\varphi }\\ M_{\varphi }\\ N_{\varphi }^{1}\\ M_{\varphi }^{2} \end{array}\right\}=\left[\begin{array}{cccc} \bar{A_{11}} & \bar{B_{11}} & \bar{C_{11}} & \bar{D_{11}}\\ \bar{B_{11}} & \bar{C_{11}} & \bar{D_{11}} & \bar{E_{11}}\\ \bar{C_{11}} & \bar{D_{11}} & \bar{E_{11}} & \bar{F_{11}}\\ \bar{D_{11}} & \bar{E_{11}} & \bar{F_{11}} & \bar{G_{11}} \end{array}\right]\left\{\begin{array}{l} \varepsilon_{\varphi }^{0}\\ k_{\varphi }^{0}\\ k_{\varphi }^{1}\\ k_{\varphi }^{2} \end{array}\right\}\left\{\begin{array}{l} Q_{\varphi }\\ P_{\varphi }\\ Q_{\varphi }^{1}\\ P_{\varphi }^{2} \end{array}\right\}=\left[\begin{array}{cccc} \bar{A_{55}} & \bar{B_{55}} & \bar{C_{55}} & \bar{D_{55}}\\ \bar{B_{55}} & \bar{C_{55}} & \bar{D_{55}} & \bar{E_{55}}\\ \bar{C_{55}} & \bar{D_{55}} & \bar{E_{55}} & \bar{F_{55}}\\ \bar{D_{55}} & \bar{E_{55}} & \bar{F_{55}} & \bar{G_{55}} \end{array}\right]\left\{\begin{array}{l} \gamma_{\varphi z}^{0}\\ k_{\varphi z}^{0}\\ k_{\varphi z}^{1}\\ k_{\varphi z}^{2} \end{array}\right\},$$

where

(12a) $$\bar{A_{ij}}=\bar{\bar{A_{ij}}}-1/R_{\varphi }\bar{\bar{B_{ij}}},\;\bar{B_{ij}}=\bar{\bar{B_{ij}}}-1/R_{\varphi }\bar{\bar{C_{ij}}}\;,\;\bar{C_{ij}}=\bar{\bar{C_{ij}}}-1/R_{\varphi }\bar{\bar{D_{ij}}},$$

(12b) $$\begin{array}{cccc} \bar{D_{ij}}=\bar{\bar{D_{ij}}}-1/R_{\varphi }\bar{\bar{E_{ij}}}, & \bar{E_{ij}}=\bar{\bar{E_{ij}}}-1/R_{\varphi }\bar{\bar{F_{ij}}}, & \bar{F_{ij}}=\bar{\bar{F_{ij}}}-1/R_{\varphi }\bar{\bar{G_{ij}}} & \bar{G_{ij}}=\bar{\bar{G_{ij}}}-1/R_{\varphi }H_{ij} \end{array},$$

(12c) $$\bar{\bar{A}}=A-BC^{-1}B^{T},$$

(12d) $$\bar{\bar{A}}=\left[\begin{array}{cccc} \bar{\bar{A_{11}}} & \bar{\bar{B_{11}}} & \bar{\bar{C_{11}}} & \bar{\bar{D_{11}}}\\ \bar{\bar{B_{11}}} & \bar{\bar{C_{11}}} & \bar{\bar{D_{11}}} & \bar{\bar{E_{11}}}\\ \bar{\bar{C_{11}}} & \bar{\bar{D_{11}}} & \bar{\bar{E_{11}}} & \bar{\bar{F_{11}}}\\ \bar{\bar{D_{11}}} & \bar{\bar{E_{11}}} & \bar{\bar{F_{11}}} & \bar{\bar{G_{11}}} \end{array}\right]A=\left[\begin{array}{cccc} A_{11} & B_{11} & C_{11} & D_{11}\\ B_{11} & C_{11} & D_{11} & E_{11}\\ C_{11} & D_{11} & E_{11} & F_{11}\\ D_{11} & E_{11} & F_{11} & G_{11} \end{array}\right],$$

(12e) $$B=\left[\begin{array}{cccccccc} A_{12} & A_{16} & B_{12} & B_{16} & C_{12} & C_{16} & D_{12} & D_{16}\\ B_{12} & B_{16} & C_{12} & C_{16} & D_{12} & D_{16} & E_{12} & E_{16}\\ C_{12} & C_{16} & D_{12} & D_{16} & E_{12} & E_{16} & F_{12} & F_{16}\\ D_{12} & D_{16} & E_{12} & E_{16} & F_{12} & F_{16} & G_{12} & G_{16} \end{array}\right],$$

(12f) $$C=\left[\begin{array}{cccc} \begin{array}{cc} A_{22} & A_{26}\\ A_{26} & A_{66} \end{array} & \begin{array}{cc} B_{22} & B_{26}\\ B_{26} & B_{66} \end{array} & \begin{array}{cc} C_{22} & C_{26}\\ C_{26} & C_{66} \end{array} & \begin{array}{cc} D_{22} & D_{26}\\ D_{26} & D_{66} \end{array}\\ \begin{array}{cc} B_{22} & B_{26}\\ B_{26} & B_{66} \end{array} & \begin{array}{cc} C_{22} & C_{26}\\ C_{26} & C_{66} \end{array} & \begin{array}{cc} D_{22} & D_{26}\\ D_{26} & D_{66} \end{array} & \begin{array}{cc} E_{22} & E_{26}\\ E_{26} & E_{66} \end{array}\\ \begin{array}{cc} C_{22} & C_{26}\\ C_{26} & C_{66} \end{array} & \begin{array}{cc} D_{22} & D_{26}\\ D_{26} & D_{66} \end{array} & \begin{array}{cc} E_{22} & E_{26}\\ E_{26} & E_{66} \end{array} & \begin{array}{cc} F_{22} & F_{26}\\ F_{26} & F_{66} \end{array}\\ \begin{array}{cc} D_{22} & D_{26}\\ D_{26} & D_{66} \end{array} & \begin{array}{cc} E_{22} & E_{26}\\ E_{26} & E_{66} \end{array} & \begin{array}{cc} F_{22} & F_{26}\\ F_{26} & F_{66} \end{array} & \begin{array}{cc} G_{22} & G_{26}\\ G_{26} & G_{66} \end{array} \end{array}\right],$$

(12g) $$\bar{\bar{Q}}=Q-SP^{-1}S^{T},$$

(12h) $$\bar{\bar{Q}}=\left[\begin{array}{cccc} \bar{\bar{A_{55}}} & \bar{\bar{B_{55}}} & \bar{\bar{C_{55}}} & \bar{\bar{D_{55}}}\\ \bar{\bar{B_{55}}} & \bar{\bar{C_{55}}} & \bar{\bar{D_{55}}} & \bar{\bar{E_{55}}}\\ \bar{\bar{C_{55}}} & \bar{\bar{D_{55}}} & \bar{\bar{E_{55}}} & \bar{\bar{F_{55}}}\\ \bar{\bar{D_{55}}} & \bar{\bar{E_{55}}} & \bar{\bar{F_{55}}} & \bar{\bar{G_{55}}} \end{array}\right]Q=\left[\begin{array}{cccc} A_{55} & B_{55} & C_{55} & D_{55}\\ B_{55} & C_{55} & D_{55} & E_{55}\\ C_{55} & D_{55} & E_{55} & F_{55}\\ D_{55} & E_{55} & F_{55} & G_{55} \end{array}\right],$$

(12i) $$S=\left[\begin{array}{cccc} A_{45} & B_{45} & C_{45} & D_{45}\\ B_{45} & C_{45} & D_{45} & E_{45}\\ C_{45} & D_{45} & E_{45} & F_{45}\\ D_{45} & E_{45} & F_{45} & G_{45} \end{array}\right]P=\left[\begin{array}{cccc} A_{44} & B_{44} & C_{44} & D_{44}\\ B_{44} & C_{44} & D_{44} & E_{44}\\ C_{44} & D_{44} & E_{44} & F_{44}\\ D_{44} & E_{44} & F_{44} & G_{44} \end{array}\right].$$

On the foundation of previous discussions, the kinetic energy of the CLCB may be represented as

(13) $$\begin{array}{l} T=\frac{1}{2}{∭}_{V}\rho \left(z\right)\left[{\left({\dot{\bar{U}}}_{ij}\right)}^{2}+{\left({\dot{\bar{W}}}_{ij}\right)}^{2}\right]\left(1+\frac{z}{R_{\varphi }}\right)R_{\varphi }d\varphi dydz=\\ =\frac{1}{2}b\int_{\varphi_{0}}^{\varphi_{1}}\left\{\begin{array}{l} I_{0}\left[{\left(\dot{u}\right)}^{2}+{\left(\dot{w}\right)}^{2}\right]+2I_{1}\dot{u}{\dot{\psi }}_{\varphi }+I_{2}\left({\dot{\psi }}_{\varphi }^{2}+2\dot{u}{\dot{\phi }}_{\varphi }\right)\\ +2I_{3}\left(\dot{u}{\dot{\lambda }}_{\varphi }+{\dot{\psi }}_{\varphi }{\dot{\phi }}_{\varphi }\right)+I_{4}\left({\dot{\phi }}_{\varphi }^{2}+2{\dot{\psi }}_{\varphi }{\dot{\lambda }}_{\varphi }\right)+2I_{5}{\dot{\phi }}_{\varphi }{\dot{\lambda }}_{\varphi }+I_{6}{\dot{\lambda }}_{\varphi }^{2} \end{array}\right\}R_{\varphi }d\varphi \end{array}$$

where

(14) $$\left(I_{0},I_{1},I_{2},I_{3},I_{4},I_{5},I_{6}\right)=\int_{z_{k}}^{z_{k+1}}\rho \left(z\right)\left(1+\frac{z}{R_{\varphi }}\right)\left(1,\;z,\;z^{2},\;z^{3},\;z^{4},\;z^{5},\;z^{6}\right)dz.$$

Besides, the strain energy may be expressed as

(15) $$U=\frac{1}{2}{∭}_{V}\left(\begin{array}{l} N_{\varphi }\varepsilon_{\varphi }^{0}+M_{\varphi }k_{\varphi }^{0}+N_{\varphi }^{1}k_{\varphi }^{1}+M_{\varphi }^{2}k_{\varphi }^{2}+\\ Q_{\varphi }\gamma_{\varphi z}^{0}+P_{\varphi }k_{\varphi z}^{0}+Q_{\varphi }^{1}k_{\varphi z}^{1}+P_{\varphi }^{2}k_{\varphi z}^{2} \end{array}\right)R_{\varphi }d\varphi dydz.$$

### 2.3. Variational Formulation for Curved Beam 

In general, by solving the governing equations associated with boundary conditions, the eigenvalue solutions can be gained. However, it is not an easy job to conduct this process, as choosing suitable admissible functions for arbitrary boundary conditions is difficult. Alternatively, a new procedure by which the problem is expressed in a modified variational form may be developed to simplify the solution. 

In this research, a highly efficient and accurate variational approach is adopted and vibration analysis of moderately tall CLCBs with diverse curvatures is conducted. First, the total variational functional $\prod_{total}$ is obtained, the terms of which include the kinetic energy *T*, strain energy *U*, and strain energy function connected with the interface and boundary restraint $\prod_{pf}$. Then, the displacement field is expanded in terms of Jacobi polynomials, which contain unknown Jacobi expanded coefficients. By conducting the variational operation (i.e., $\delta \prod_{total}=0$) with respect to unknown Jacobi expanded coefficients, an equation in matrix form can be acquired. Through solving this equation, the frequencies and corresponding mode shapes can be easily determined.

Besides, the formulation is derived by employing the multi-segment partitioning technique. The curved beam is separated into *N_φ_* identical segments along the *φ* orientation. As each segment becomes a free–free substructure, the displacement discretization with respect to admissible functions becomes more convenient. This is related to the fact that the continuity conditions of the CLCB need not be imposed, as their satisfaction is implemented in a variational statement. As such, the issue is simplified to simulating the interaction of each beam segment with common boundaries. This will make the computer implementation of CLCBs much simpler: (a) The selections of admissible functions become flexible as both boundary and continuity conditions are relaxed; (b) by flexibly choosing the appropriate admissible functions, the convergence performance of the present methodology can be quite fast and stable; and (c) the accuracy of the modified variational method can be substantially improved by segmentation, especially for higher modes. Then, the vibration problem may be distinguished by an improved variational principle [24,25,26], which turns into finding the minimum value of a variational functional as

(16) $$\prod_{ve}=\sum_{i=1}^{N_{\varphi }}\left(U_{i}-T_{i}\right),$$

where *T_i_* is the maximum kinetic energy of the *i*’th beam segment and *U_i_* is the corresponding maximum strain energy. 

To construct a uniform model to handle arbitrary boundary conditions, as well as ensure numerical stability, the technique of penalty function is exploited. The strain energy function connected with the boundary elastic restraint can be shown as

(17) $$\begin{array}{l} \prod_{pf}=\frac{1}{2}\sum_{i=1}^{N_{\varphi }}\left[\begin{array}{l} \eta_{u}k_{u}{\left(u_{i}-u_{i+1}\right)}^{2}+\eta_{w}k_{w}{\left(w_{i}-w_{i+1}\right)}^{2}+\eta_{\varphi }k_{\varphi }{\left(\varphi_{i}-\varphi_{i+1}\right)}^{2}\\ +\eta_{\phi }k_{\phi }{\left(\phi_{i}-\phi_{i+1}\right)}^{2}+\eta_{\nu }k_{\nu }{\left(\nu_{i}-\nu_{i+1}\right)}^{2} \end{array}\right]\\ =\frac{1}{2}{\left[k_{u0}u^{2}+k_{w0}w^{2}+k_{\varphi 0}\varphi^{2}+k_{\phi 0}\phi^{2}+k_{\nu 0}\nu^{2}\right]}_{\varphi =\varphi_{0}}\\ +\frac{1}{2}{\left[k_{u1}u^{2}+k_{w1}w^{2}+k_{\varphi 1}\varphi^{2}+k_{\phi 1}\phi^{2}+k_{\nu 1}\nu^{2}\right]}_{\varphi =\varphi_{1}}\\ +\frac{1}{2}\sum_{i=1}^{N_{\varphi }}\left[\begin{array}{l} k_{u}{\left(u_{i}-u_{i+1}\right)}^{2}+k_{w}{\left(w_{i}-w_{i+1}\right)}^{2}+k_{\varphi }{\left(\varphi_{i}-\varphi_{i+1}\right)}^{2}\\ +k_{\phi }{\left(\phi_{i}-\phi_{i+1}\right)}^{2}+k_{\nu }{\left(\nu_{i}-\nu_{i+1}\right)}^{2} \end{array}\right] \end{array}$$

where $k_{\tau }\left(\tau =u,w,\varphi ,\phi ,\nu \right)$ indicates the penalty terms expressing the elastic stiffness at both ends of the curved beam, and $\eta_{\tau }\left(\tau =u,w,\varphi ,\phi ,\nu \right)$ represents the continuity or boundary coefficients. By properly defining $\eta_{\tau }$ and $k_{\tau }$, both continuity conditions for the interface and the arbitrary boundary conditions can be conveniently obtained. For the continuity conditions of two neighbored curved beam domains, the continuity coefficients $\eta_{\tau }\left(\tau =u,w,\varphi ,\phi ,\nu \right)=1$, while for boundary conditions, different combinations of boundary coefficients $\eta_{\tau }$ and $k_{\tau }$ are imposed to achieve various boundary conditions (Table 1). Meanwhile, it is worth noting that both classical and elastic boundary restraints can be achieved by properly selecting the values of $k_{\tau }\left(\tau =u,w,\varphi ,\phi ,\nu \right)$; note that $k_{\tau 0}$ and $k_{\tau 1}$ represent the penalty terms at edges $\varphi =\varphi_{0}$ and $\varphi =\varphi_{1}$, respectively. The elastic boundary restraints represent a boundary condition between simply supported and clamped boundary conditions, which can be modeled by springs at the edges. For instance, by setting one or some of the $k_{\tau }\left(\tau =u,w,\varphi ,\phi ,\nu \right)$ at certain values, the elastic boundary conditions can be conveniently obtained. 

More information about the penalty terms to handle the boundary conditions can be found in previous investigations [27,28,29,30,31]. Consequently, the variational form for a CLCB subjected to arbitrary boundary conditions is

(18) $$\prod_{total}=\sum_{i=1}^{N_{\varphi }}\left(U_{i}-T_{i}\right)+\prod_{pf}.$$

### 2.4. Solution Procedure

By introducing the modified variational approach in conjunction with the multi-segment partitioning strategy, the choice of admissible displacements for each beam segment can be flexible. This is due to the fact that continuity and boundary conditions of the curved beam are relaxed by the functional $\prod_{total}$. The $\prod_{total}$ allows the use of identical displacement functions for every curved beam segment. In the present analysis, the displacement components for each divided segment of the CLCB are congruously represented by means of Jacobi polynomials.

(19a) $$u^{i}=\sum_{m=0}^{M}U_{m}P_{m}^{\left(\alpha ,\beta \right)}(\varphi )e^{i\omega t},$$

(19b) $$w^{i}=\sum_{m=0}^{M}W_{m}P_{m}^{\left(\alpha ,\beta \right)}(\varphi )e^{i\omega t},$$

(19c) $$\psi^{i}{}_{\varphi }=\sum_{m=0}^{M}\psi_{\varphi_{m}}P_{m}^{\left(\alpha ,\beta \right)}(\varphi )e^{i\omega t},$$

(19d) $$\phi^{i}{}_{\varphi }=\sum_{m=0}^{M}\Phi_{\varphi_{m}}P_{m}^{\left(\alpha ,\beta \right)}(\varphi )e^{i\omega t},$$

(19e) $$\lambda^{i}{}_{\varphi }=\sum_{m=0}^{M}{ϒ}_{\varphi_{m}}P_{m}^{\left(\alpha ,\beta \right)}(\varphi )e^{i\omega t},$$

where $U_{m}$, $W_{m}$, $\psi_{\varphi_{m}}$, $\Phi_{\varphi_{m}}$, and ${ϒ}_{\varphi_{m}}$ indicate the relevant Jacobi expanded coefficients; $P_{m}^{\left(\alpha ,\beta \right)}(\varphi )$ represents the Jacobi polynomial of order *m*, which is related to the displacement components along the central line orientation; and ω and *t* signify angular frequency and time, respectively. Besides, the maximum value of *m* or the truncation terms are represented by *M*. By choosing different combinations of Jacobi parameters *α* and *β*, various orthogonal polynomials can be obtained such as Chebyshev, Gegenbauer, and Legendre polynomials. More details about these can be discovered in [32,33,34]. Substituting Equations (13) and (15)–(17) into Equation (18) and incorporating Equation (19), the following can be achieved:

(20) $$\prod_{total}=\prod_{total}\left(U_{m},V_{m},\psi_{\varphi_{m}},\Phi_{\varphi_{m}},{ϒ}_{\varphi_{m}}\right).$$

Conducting the variation operation for $\prod_{total}$, i.e., $\delta \prod_{total}=0$, with regard to Jacobi expanded coefficients ($U_{m}$, $V_{m}$, $\psi_{\varphi_{m}}$, $\Phi_{\varphi_{m}}$, and ${ϒ}_{\varphi_{m}}$), the following characteristic equations can be obtained:

(21) $$\left(K-\omega^{2}M\right)E=0,$$

in which **K** signifies the stiffness matrix, **M** indicates the mass matrix, and **E** denotes the undetermined coefficients. Apparently, through the solution of Equation (21), the eigenvalues and eigenvectors can be acquired. 

## 3. Results and Discussions

In this part, the convergence performance, reliability, efficiency, and accuracy of the current methodology are studied by a number of numerical cases. For the sake of brevity, the free, clamped, simply supported, slided, and elastic boundary conditions are represented by F, C, SS, SD, and E, respectively, as shown in Table 1. Besides, three kinds of elastic boundary restraints (E1, E2, and E3) are taken into consideration. Then, a two-letter string is utilized to represent the boundary conditions of two ends, e.g., SS-E indicates the curved beam subjected to the simply supported boundary condition at edge *φ* = *φ*_0_ and the elastic one at edge *φ* = *φ*_1_. 

First, it is essential to investigate the convergence performance of the present modified variational approach for vibration analysis of the curved beam. To begin with, the comparison of dimensionless frequencies Ω for a C-C circular CLCB with respect to different *N_φ_* is presented in Table 2. The non-dimensional frequency Ω is defined as $\Omega =\omega \left(R^{2}{\left(\varphi_{1}-\varphi_{0}\right)}^{2}\right)\sqrt{12\rho /\left(E_{1}h^{2}\right)}$. Two thicknesses *h* = 0.1 and 0.2 m are considered. The material properties are *ρ* = 1580 kg/m^3^, *E*_2_ = 10 GPa, *E*_1_ = 15*E*_2_, *G*_12_ = *G*_13_ = *G*_23_ = 0.55*E*_2_, *μ*_12_ = 0.27, and $[\alpha_{fiber}^{1}/\alpha_{fiber}^{2}]=\left[0{}^{\circ}/90{}^{\circ}\right]$ (i.e., the fiber orientation angles of the first and second lamina are 0° and 90°, respectively). In addition, the geometric parameters are *φ*_0_ = −π/3 and *φ*_1_ = π/3. The Jacobi parameters are *α* = *β* = 0 and the truncation number is *M* = 8. The number of segments is selected as *N_φ_* = 2, 4, 6, 8, and 10. In addition, the results from exact solutions exploiting CBT [3] and exact serious results based on FSDT [14] are shown for comparison. As *N_φ_* is increased, fast convergence of Ω can be observed. In addition, the present results coincide well with the ones by FSDT and CBT. As a consequence, with small *N_φ_*, accurate results of Ω can be achieved. For subsequent cases, unless otherwise specified, *N_φ_* = 8 is chosen by default. 

**Figure 3:**
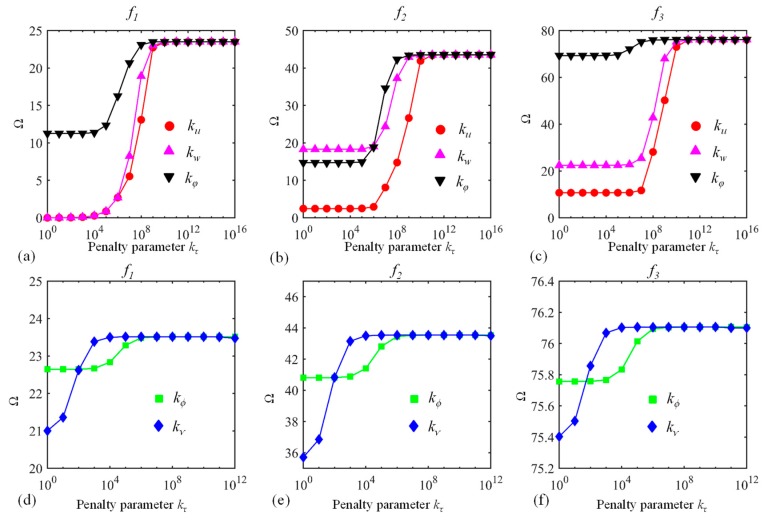
Dimensionless frequency Ω versus penalty parameters $k_{\tau }$: (**a**–**c**) $k_{\tau }\left(\tau =u,w,\varphi \right)$ and (**d**–**f**) $k_{\tau }\left(\tau =\phi ,\nu \right)$. The first three modes (*f*_1_, *f*_2_, and *f*_3_) are considered.

Secondly, the influence of penalty terms $k_{\tau }\left(\tau =u,w,\varphi ,\phi ,\nu \right)$ (see Equation (19)) on Ω is investigated. The dimensionless frequency Ω versus penalty parameters *k_t_* for a C-C curved beam is shown in Figure 3. The free vibration of CLCBs with arbitrary boundary conditions including both classical (clamped, free, and simply supported boundary conditions) and elastic boundary conditions is investigated. Here, for the convergence performance of penalty terms, the typical C-C boundary conditions are selected. Note that for other boundary conditions, similar results can be achieved. The material properties, geometric parameters, Jacobi parameters (*α* and *β*), and number of truncation terms *M* are the same as those of Table 2. For each case, one type of $k_{\tau }$ is varied from 10^0^ to 10^16^ while the others are unchanged (= 10^14^). When penalty parameters are absent or small, pseudo-rigid modes might emerge, indicating that the continuity conditions of the interface may not be imposed properly. By augmenting $k_{\tau }$, the continuity condition can be satisfied. It is observed that when $k_{\tau }$ is in the range of 10^10^–10^16^, the solution becomes very stable, with Ω remaining the same. Therefore, $k_{\tau \left(u,v,\varphi \right)}=10^{14}$ and $k_{\tau \left(\phi ,\nu \right)}=10^{12}$ are employed as the coupling parameters in the following discussions. It should be noted that for different types of curved beams (e.g., elliptical, paraboloidal, and hyperbolical ones), when $k_{\tau \left(u,v,\varphi \right)}$ > 10^10^ and $k_{\tau \left(\phi ,\nu \right)}$ > 10^8^, similarly, Ω converges to certain values (not shown). Thus, the determinations of penalty and boundary parameters are consistent for different curved beams.

**Figure 4:**
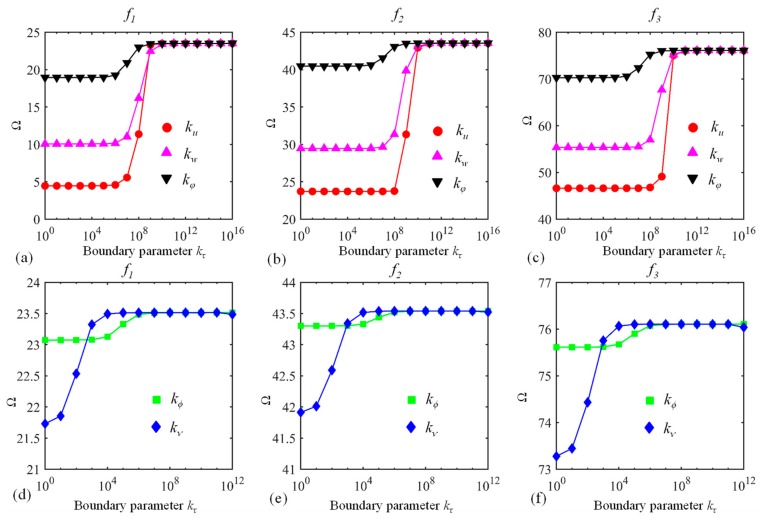
Dimensionless frequency Ω versus boundary parameters $k_{\tau }$: (**a**–**c**) $k_{\tau }\left(\tau =u,w,\varphi \right)$ and (**d**–**f**) $k_{\tau }\left(\tau =\phi ,\nu \right)$. The first three modes (*f*_1_, *f*_2_, and *f*_3_) are considered.

It has been mentioned before that the present approach can be applicable to the prediction of the vibration behavior of a curved laminated beam subjected to elastic boundary conditions. Hence, the impact of boundary parameters on the vibration characteristics should be analyzed. The dimensionless frequency Ω versus boundary parameters $k_{\tau }$ for a C-E curved laminated beam is shown in Figure 4, with the other parameters being the same as those of Figure 3. The edge *φ* = *φ*_0_ is clamped while the border *φ* = *φ*_1_ is elastically supported. For the edge *φ* = *φ*_1_, one set of boundary springs vary between 10^0^ and 10^16^ while the others are presumed to be infinity (= 10^14^). The variations of Ω with $k_{\tau }$ are similar to those in Figure 3, and the results can reflect that the determination of $k_{\tau }$ in Table 1 for different boundary conditions should be appropriate. 

**Figure 5:**
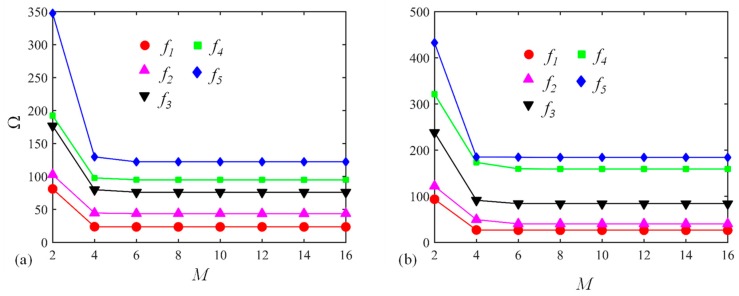
Dimensionless frequency Ω versus truncation terms *M* for (**a**) a circular CLCB and (**b**) an elliptical CLCB with C-C boundary condition.

**Figure 6:**
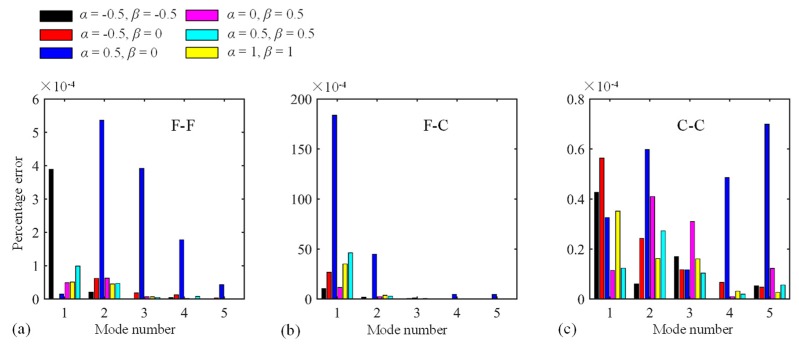
Percentage error of Ω for diverse combinations of *α* and *β*. (**a**)F-F boundary condition; (**b**) F-C boundary condition; (**c**) C-C boundary condition

Thirdly, the Jacobi orthogonal polynomials including the number of truncation terms and Jacobi parameters (*α* and *β*) should be investigated. The dimensionless frequency Ω versus truncation terms *M* for a circular CLCB and elliptical CLCB with the C-C boundary condition is displayed in Figure 5a,b, respectively, the other parameters remaining the same as those of Figure 3. Obviously, for both cases, Ω converges very fast with the increase in *M*. As *M* becomes larger than 8, Ω (first five modes) converges to certain values. Subsequently, *M* = 8 is selected for all the numerical examples. To demonstrate the effect of Jacobi parameters (*α* and *β*) on the vibration behavior of the curved beam, the percentage error of Ω for various combinations of *α* and *β* is illustrated in Figure 6. Several combinations of (*α*, *β*) including (*α*, *β*) = (−0.5, −0.5), (−0.5, 0), (0.5, 0), (0, 0.5), (0.5, 0.5), and (1, 1) are chosen. The results of (*α*, *β*) = (0, 0) are used as the base results, with the percentage error defined as $\left(\Omega_{\alpha ,\beta }-\Omega_{\alpha =0,\beta =0}\right)/\Omega_{\alpha =0,\beta =0}$. In addition, three types of boundary restraints are considered: F-F, F-C, and C-C boundary conditions. It is shown that the maximum value of the percentage error is no more than 2 × 10^−2^, which may indicate that the Jacobi parameters may not affect the value of Ω. Thus, different admissible displacement functions with various Jacobi parameters can be used to establish the formulation, leading to flexible selections of admissible displacements. For the following calculations, unless otherwise stated, (*α*, *β*) = (0, 0) is utilized.

**Figure 7:**
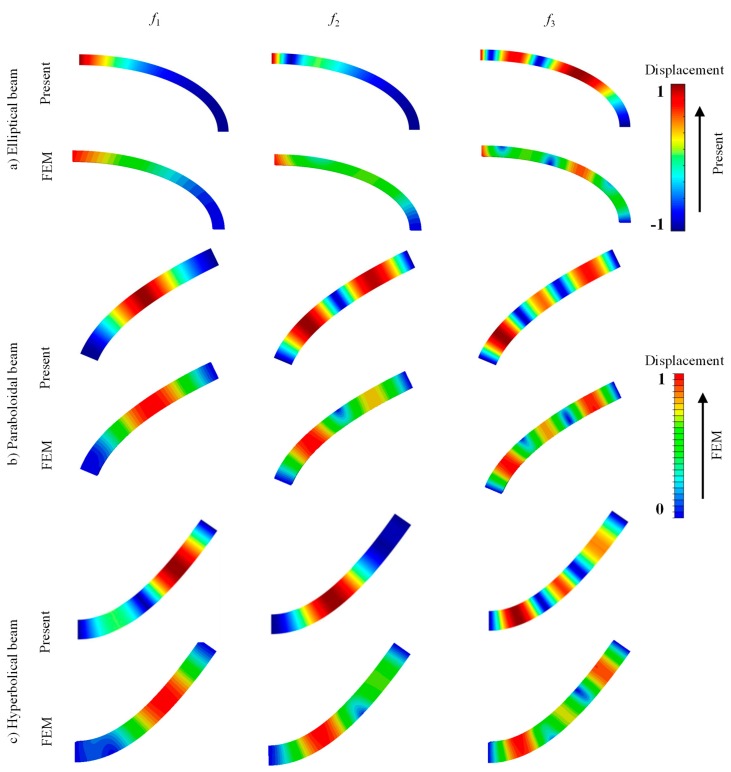
Several mode shapes in *x–z* plane for CLCBs: (**a**) Elliptical beam, (**b**) paraboloidal beam, and (**c**) hyperbolical beam.

After comprehending the characteristics of several parameters, it is necessary to show the reliability and precision of the present approach. Due to lack of experimental data, the present results are compared to those from results by other numerical studies in the literature. To further validate the present method, the finite element method (FEM) is also utilized for comparison. Table 3 shows the comparison of Ω (first eight modes) for the C-C elliptical, paraboloidal, and hyperbolical CLCBs. The material parameters are *ρ* = 1500 kg/m^3^, *E*_2_ = 10 GPa, *E*_1_ = 15*E*_2_, *G*_12_ = *G*_13_ = 0.5*E*_2_, *G*_23_ = 0.6*E*_2_, *μ*_12_ = 0.25, and $\alpha_{fiber}^{k}=\left[0{}^{\circ}/90{}^{\circ}/0{}^{\circ}\right]$. The geometric dimensions are: (a) **Elliptical one:** *h* = 0.15 m, *a_e_* = 2 m, *b_e_* = 1 m, *φ*_0_ = 0, and *φ*_1_ = π/2; (b) **Paraboloidal one:** *h* = 0.15 m, *L* =1 m, *R*_0_ = 0.2 m, and *R*_1_ = 1 m; **Hyperbolical one:** *a_h_* = 3 m, *h* = 0.15 m, *R*_0_ = 0.2 m, *R*_1_ = 1 m, *R*_s_ = 4 m, and *L* = 1 m. Free vibration results obtained through the finite element method (FEM) using ABAQUS software are given as a reference. The types of elements are quadrilateral S4R (S4R is a general shell element type in ABAQUS) for all the cases. The numbers of elements for the elliptical, paraboloidal, and hyperbolical beams are 5808, 1310, and 1330, respectively. As shown in Table 3, the errors of the results by the present method and those by the FEM are not larger than 2.92%, which is small and indicates the accuracy of the present methodology. The natural frequencies depend on the different radii of curvature, lamination schemes, and boundary conditions. The differences of frequencies at the C-C boundary condition with respect to different types of CLCB (i.e., elliptical, paraboloidal, and hyperbolical) should be related to the radii of curvature changes. Then, the comparison of Ω for circular CLCBs subjected to various boundary conditions is displayed in Table 4. Results from the decomposition approach [17] and wave solution method [35] are given as a contrast. Three boundary restraints F-F, F-C, and C-SS are taken into account. For all the cases, the frequencies are in complete agreement. To further validate the present method, several mode shapes for curved composite laminated beams including elliptical, paraboloidal, and hyperbolical ones in the *x–z* plane are presented in Figure 7. The mode shapes obtained by the FEM through ABAQUS are presented for comparison. Apparently, all the mode shapes from the present methodology and FEM match well with each other. On the whole, the present methodology is capable of solving the vibration problem of the CLCB subjected to arbitrary boundary conditions. 

Finally, several novel results are presented, which can be served as benchmark solutions. The frequencies (first five modes) for the elliptical, paraboloidal, and hyperbolical CLCB with diverse lamination schemes and boundary conditions are shown in Table 5, Table 6 and Table 7, respectively. For each kind of beam, four lamination schemes including $\alpha_{fiber}^{k}$ = [0°], [0°/90°], [0°/90°/0°], and [0°/90°/0°/90°] are considered. Besides, four types of classical (C-C, SS-SD, F-C, and F-F) and three kinds of elastic boundary restraints (E1-E1, E2-E2, and E3-E3) are studied. The geometric parameters are: (a) **Elliptical one:** *h* = 0.2 m, *b_e_* = 1 m, *a_e_* = 2 m, *φ*_0_ = −π/3, and *φ*_1_ = π/3; (b) **Paraboloidal one:** *h* = 0.2 m, *L* = 2 m, *R*_0_ = 0.2 m, and *R*_1_ = 1 m; and (c) **Hyperbolical one:** *h* = 0.2 m, *a_h_* = 3 m, *R*_s_ = 4 m, *R*_0_ = 0.5 m, *R*_1_ = 0.5 m, *L* = 2 m, and *L*_1_ = 1 m. Several aspects should be pointed out in Table 5, Table 6 and Table 7. First, the natural frequencies depend on the different radii of curvature, lamination schemes, and boundary conditions. Secondly, despite different radii of curvature and boundary conditions, the fundamental frequencies of the CLCB that own a single layer ([0°]) or symmetric lamination [0°/90°/0°] are always those that have anti-symmetric laminations ([0°/90°] and [0°/90°/0°/90°]. Thirdly, the C-C boundary condition always corresponds to the largest fundamental frequencies, regardless of the radii of curvature and lamination schemes. 

**Figure A1:**
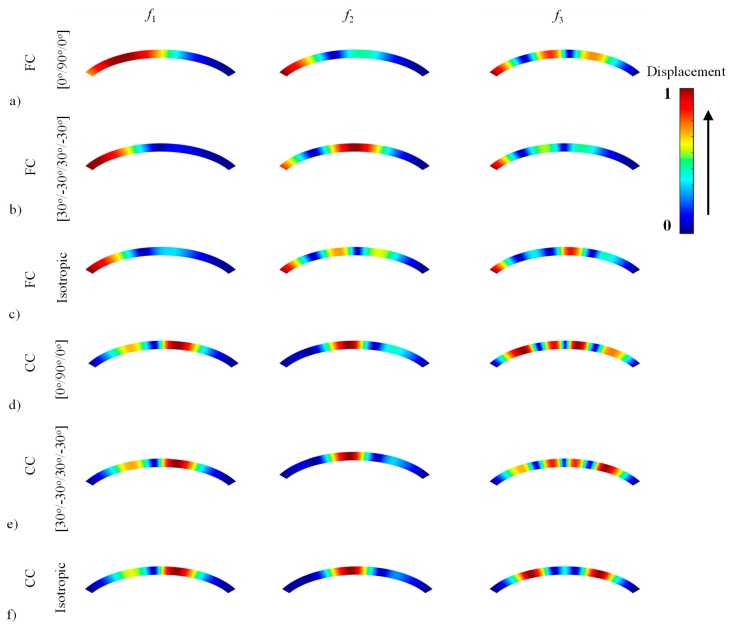
Several mode shapes for an elliptical composite laminated beam and an elliptical isotropic beam.

**Figure A2:**
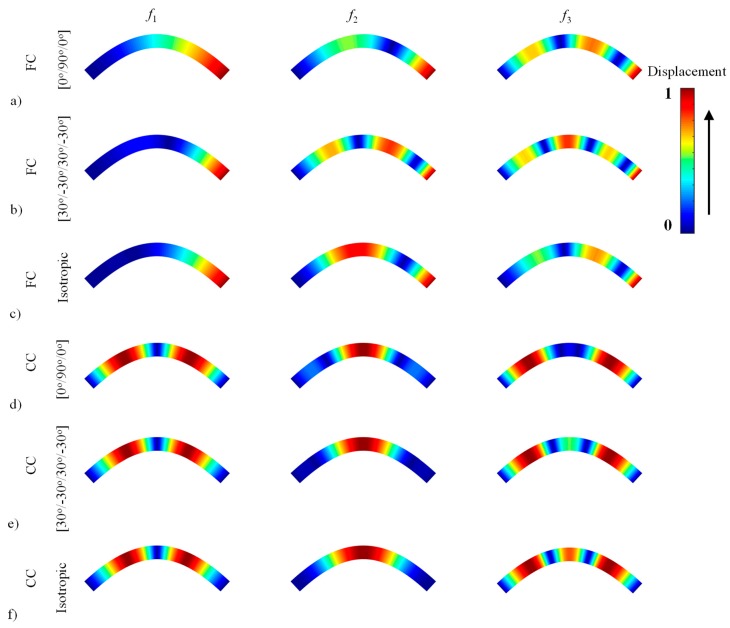
Several mode shapes for a paraboloidal composite laminated beam and a paraboloidal isotropic beam.

**Figure A3:**
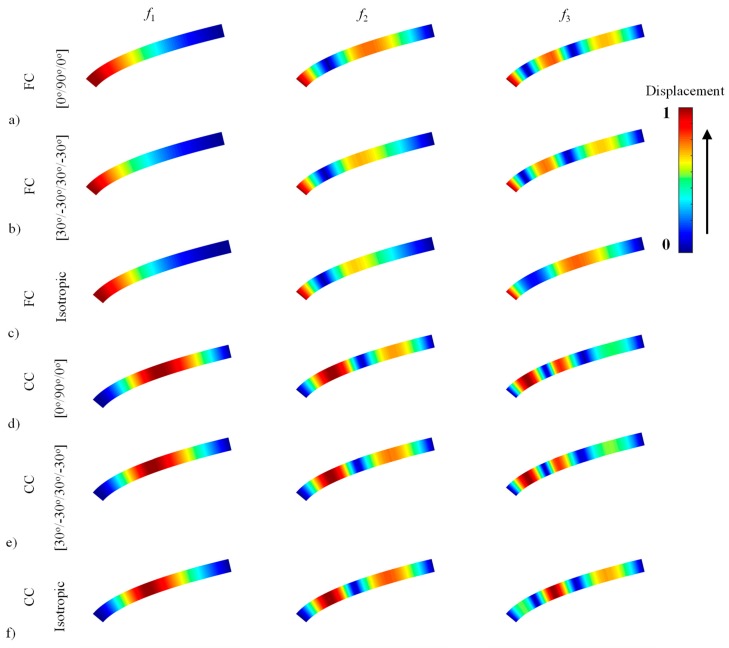
Several mode shapes for a hyperbolical composite laminated beam and a hyperbolical isotropic beam.

Several mode shapes of elliptical (Figure A1), paraboloidal (Figure A2), and hyperbolical (Figure A3) CLCBs with different lamination schemes ([0°/90°/0°] and [30°/−30°/30°/–30°]) and boundary conditions (C-F and C-C) are presented (see the Appendix A). The material and geometric properties in Figure A1 and Figure A2 are in accordance with those in Table 5, Table 6 and Table 7, respectively. To demonstrate the material properties, the mode shapes of isotropic beams (elliptical, paraboloidal, and hyperbolical) are also given in Figure A1, Figure A2 and Figure A3. The material properties of the isotropic beams are *ρ* = 1500 kg/m^3^, *E* = 100 GPa, and *μ* = 0.25. The following aspects should be noted. First, for all the cases, the mode shapes for laminated composite beams and isotropic beams are different. With regard to the F-C boundary condition, obvious differences can be observed for the first-order mode, while for the C-C boundary condition, the mode shapes are similar for the first- and second-order modes, although distinct differences can be observed for the third-order mode. Secondly, with various radii of curvature changes and boundary conditions, the corresponding mode shapes are also quite different. Thirdly, by choosing different lamination schemes, the material properties change, leading to variations in mode shapes of the CLCBs. By properly selecting the lamination schemes, high strength ratio and corrosion resistance can be obtained, which may make the laminated composite materials perform better than others.

## 4. Conclusions

This paper proposes a unified formulation for dynamic analysis of different types of CLCBs subjected to general boundary conditions. In the framework of HSDT, an improved variational approach along with a multi-segment partitioning technique is exploited to construct the theoretical model. The displacement components of each curved beam segment are presented in terms of Jacobi orthogonal polynomials. Through a series of numerical cases, the fast convergence performance and stable, highly efficient, and precise features of the current methodology have been demonstrated. It has been proved that the present methodology can be applicable for vibration cases with respect to both classic and elastic boundary conditions. Furthermore, it should be noted that the current approach leads to flexible choices of admissible displacement functions, which is one prominent advantage compared to other methods. This paper presents the free vibration results, while the dynamic analysis of CLCBs under external excitation by the present approach has not been studied. Besides, linear geometric analysis of CLCBs by a unified formulation has been conducted. The present approach should also be appropriate for non-linear geometric analysis of CLCBs by considering the non-linear geometric effect (e.g., introducing the damping force). These will be considered in our future study.

## Figures and Tables

**Table 1:** Boundary coefficients and penalty parameters for various boundary conditions.

Boundary Conditions	Boundary Coefficients					Penalty Parameters				
	*η_u_*	*η_w_*	*η_φ_*	*η_ϕ_*	*η_ν_*	*k_u_*	*k_w_*	*k_φ_*	*k_ϕ_*	*k_ν_*
Free (F)	0	0	0	0	0	0	0	0	0	0
Simply supported (SS)	1	1	0	0	0	10^14^	10^14^	0	0	0
Slided (SD)	0	1	0	0	0	0	10^14^	0	0	0
Clamped (C)	1	1	1	1	1	10^14^	10^14^	10^14^	10^8^	10^8^
Elastic supported 1 (E1)	1	1	1	1	1	10^8^	10^8^	10^14^	10^8^	10^8^
Elastic supported 1 (E2)	1	1	1	1	1	10^14^	10^14^	10^8^	10^8^	10^8^
Elastic supported 1 (E3)	1	1	1	1	1	10^8^	10^8^	10^8^	10^8^	10^8^

**Table 2:** Comparison of dimensionless frequencies Ω (first five modes) for a C-C curved laminated composite beam with respect to different *N_φ_*.

*h*	Mode Number	Number of the Segment *N_φ_*					Ref. [14]	Ref. [3]
		2	4	6	8	10		
0.1	1	23.519	23.514	23.514	23.513	23.513	23.245	23.628
	2	43.556	43.540	43.537	43.538	43.536	42.803	43.800
	3	76.148	76.105	76.101	76.099	76.099	74.476	76.687
	4	94.982	94.936	94.929	94.925	94.921	93.286	95.388
	5	122.318	122.192	122.186	122.183	122.180	120.064	123.059
0.2	1	19.816	19.805	19.803	19.799	19.795	19.116	20.005
	2	32.761	32.736	32.724	32.714	32.703	31.450	33.003
	3	55.356	55.340	55.304	55.264	55.224	52.850	55.849
	4	55.495	55.480	55.454	55.446	55.437	54.094	55.971
	5	79.636	79.568	79.550	79.544	79.533	75.675	80.422

**Table 3:** Comparison of frequencies (first eight modes) for C-C elliptical, paraboloidal, and hyperbolical curved laminated composite beams.

Mode No.	Elliptical Beam			Paraboloidal Beam			Hyperbolical Beam		
	Present	FEM	Error (%)	Present	FEM	Error (%)	Present	FEM	Error (%)
1	380.16	376.6	0.95	739.05	734	0.68	793.17	784.9	1.05
2	594.22	589.8	0.75	1162.5	1155.3	0.62	1207.4	1195.3	1.01
3	934.78	930.1	0.50	1826.5	1807.7	1.03	1809.0	1773.4	2.01
4	1116.0	1111.4	0.41	2481.2	2449.6	1.27	2433.8	2375.3	2.46
5	1503.5	1490.6	0.87	3161.2	3110.7	1.60	3107.4	3042	2.15
6	1819.4	1813.8	0.31	3409.7	3391	0.55	3451.1	3443.1	0.23
7	2169.6	2151.3	0.85	4011.5	3915.2	2.40	3979.0	3866	2.92
8	2375.5	2359.8	0.67	4655.6	4582	1.58	4565.8	4498.2	1.50

**Table 4:** Comparison of Ω (first five modes) for circular curved laminated beams subjected to various boundary conditions.

Mode No.	F-F			F-C			C-SS		
	HBT_[LST]_	HBT_[LMR]_	Present	HBT_[LST]_	HBT_[LMR]_	Present	HBT_[LST]_	HBT_[LMR]_	Present
1	765.68	765.306	771.373	338.33	338.197	343.494	764.78	763.348	769.884
2	2100.96	2097.346	2115.244	1349.00	1346.992	1341.057	2097.01	2088.465	2108.493
3	4092.08	4077.308	4115.793	3019.38	3009.457	3093.753	4081.73	4053.791	4097.477
4	6707.87	6666.978	6744.373	5329.25	5298.837	5384.977	6686.94	6619.074	6700.256

**Table 5:** Frequencies (first five modes) for an elliptical curved laminated composite beam with diverse lamination schemes and boundary conditions.

Lamination Schemes (°)	*f* (Hz)	Boundary Conditions						
		F-F	F-C	SS-SD	C-C	E1-E1	E2-E2	E3-E3
[0]	1	103.35	18.207	21.931	214.16	56.997	171.69	53.464
	2	262.64	85.296	150.65	263.09	61.286	254.28	59.808
	3	460.62	226.56	300.36	539.35	97.939	512.11	97.190
	4	677.35	384.05	505.66	583.42	193.52	580.83	177.09
	5	898.48	581.31	685.43	886.62	359.43	860.50	320.60
[0/90]	1	51.865	8.846	11.239	128.84	48.745	112.12	46.682
	2	141.13	45.614	82.267	174.40	59.679	167.82	58.532
	3	267.66	129.77	179.62	371.09	83.032	348.96	82.089
	4	422.68	240.35	324.54	397.31	130.95	393.14	130.60
	5	597.93	378.64	469.09	633.42	220.11	586.10	213.05
[0/90/0]	1	102.10	17.923	21.640	216.71	56.984	172.22	53.410
	2	262.42	84.886	150.17	254.95	61.292	248.28	59.810
	3	465.29	227.31	300.04	547.94	97.685	518.58	97.038
	4	690.84	383.60	506.01	550.78	193.14	539.92	176.80
	5	920.99	576.64	653.50	867.54	362.43	833.45	322.23
[0/90/0/90]	1	72.274	12.514	15.476	167.75	53.351	138.49	50.262
	2	192.11	62.109	110.87	208.30	60.627	201.28	59.339
	3	353.63	172.04	232.27	450.34	89.398	423.16	89.277
	4	542.19	303.71	406.54	454.42	155.25	445.22	150.87
	5	744.91	464.35	547.68	729.13	280.43	687.52	260.79

**Table 6:** Frequencies (first five modes) for a paraboloidal curved laminated composite beam with diverse lamination schemes and boundary conditions.

Lamination Schemes (°)	*f* (Hz)	Boundary Conditions						
		F-F	F-C	SS-SD	C-C	E1-E1	E2-E2	E3-E3
[0]	1	373.52	64.623	164.28	371.90	85.164	322.36	82.579
	2	800.98	312.45	507.90	657.91	87.915	607.76	87.789
	3	1253.6	707.78	881.86	1036.3	210.21	997.29	175.46
	4	1714.7	1110.7	1167.5	1424.0	552.63	1401.6	464.38
	5	2242.4	1181.7	1455.7	1859.1	964.69	1843.9	867.60
[0/90]	1	197.02	32.159	87.238	245.62	81.521	197.89	77.791
	2	491.20	185.02	320.31	469.30	87.631	420.62	87.597
	3	857.49	468.35	641.00	790.40	149.76	710.61	147.49
	4	1275.6	817.64	769.92	1149.3	349.65	1105.4	321.28
	5	1593.7	834.84	1048.4	1518.4	667.48	1415.6	612.78
[0/90/0]	1	373.15	63.977	163.75	358.74	85.290	300.87	82.653
	2	817.30	316.41	512.27	664.06	87.895	602.30	87.767
	3	1292.5	725.21	830.90	1058.7	209.82	1013.9	175.42
	4	1775.5	962.32	1061.6	1460.7	562.24	1437.0	468.36
	5	1921.2	1169.0	1464.0	1848.6	994.58	1841.3	888.14
[0/90/0/90]	1	270.67	45.008	119.10	290.34	83.809	237.39	80.442
	2	635.86	242.33	406.68	554.66	87.796	496.35	87.709
	3	1055.0	583.18	731.14	905.41	172.60	841.77	161.38
	4	1498.5	844.42	897.74	1277.4	442.01	1249.1	385.83
	5	1673.8	974.15	1237.1	1626.3	813.80	1588.1	733.36

**Table 7:** Frequencies (first five modes) for a hyperbolical curved laminated composite beam with diverse lamination schemes and boundary conditions.

Lamination Schemes (°)	*f* (Hz)	Boundary Conditions						
		F-F	F-C	SS-SD	C-C	E1-E1	E2-E2	E3-E3
[0]	1	304.37	63.190	84.575	516.70	83.783	454.97	82.037
	2	706.30	215.16	437.29	896.56	84.652	867.29	82.866
	3	1133.2	599.33	851.70	1121.3	201.41	1098.4	169.28
	4	1564.2	1018.0	1265.3	1308.2	457.61	1284.3	390.81
	5	1987.4	1414.5	1411.8	1718.8	862.73	1701.3	772.01
[0/90]	1	166.56	31.709	46.314	375.75	81.434	331.10	78.948
	2	446.82	132.13	279.03	689.45	82.951	647.32	82.043
	3	776.96	399.38	585.56	834.51	143.64	739.01	141.75
	4	1162.2	743.98	878.91	1057.0	303.53	999.08	283.67
	5	1537.4	1058.2	988.53	1429.0	608.67	1378.6	561.35
[0/90/0]	1	303.83	62.500	84.171	531.51	83.853	462.71	82.065
	2	719.58	217.47	442.46	881.36	84.665	877.44	82.869
	3	1165.7	610.38	876.96	991.48	200.91	931.96	169.20
	4	1620.2	1050.2	1103.1	1356.6	464.23	1329.0	393.67
	5	2061.9	1277.5	1316.8	1782.3	887.96	1763.4	789.18
[0/90/0/90]	1	225.23	44.300	62.334	446.17	82.850	388.20	80.604
	2	570.08	169.93	352.15	766.94	83.946	757.90	82.550
	3	955.03	493.94	721.36	864.92	165.59	781.68	155.42
	4	1371.1	882.44	1018.6	1184.5	373.57	1146.2	332.75
	5	1760.7	1113.6	1118.4	1572.6	734.99	1541.4	663.31
